# Design and Fabrication of Highly Porous Replicated Aluminum Foam Using Double-Granular Space Holder

**DOI:** 10.3390/ma14071619

**Published:** 2021-03-26

**Authors:** Arkady Finkelstein, Dmitry Husnullin, Konstantin Borodianskiy

**Affiliations:** 1 Department of Foundry Engineering and Strengthening Technologies, Ural Federal University, 620002 Yekaterinburg, Russia; avinkel@mail.ru; 2 Composite Materials Ltd., 624140 Kirovgrad, Russia; barbados1991@yandex.ru; 3 Department of Chemical Engineering, Ariel University, Ariel 40700, Israel

**Keywords:** replicated foam, Al alloy, porosity, vacuum casting, space holder, packing density model

## Abstract

Porous materials are widely employed in a wide variety of industrial applications due to their advanced functional performance. Porous aluminum is among the most attractive metallic materials. It can be produced using repeatable methods involving a replicated Al foam that also provides porosity control. In this work, a highly porous replicated Al foam was fabricated. First, the model of multifunctional packing density was used and corrected to select the appropriate space holders. Then, Al foam was produced using a double-granular sodium chloride space holder. The obtained results showed a maximum porosity of 65% that was achieved using a mix of coarse, irregular granules with spherical granules of intermediate size.

## 1. Introduction

Porosity is one of the most important parameters in the processing and production of porous materials. It affects the physical properties of materials such as their thermal and electrical conductivity, different mechanical properties, and functional performance (e.g., permeability, sound absorption ability, fluids filtration, damping capacity, etc.) [1,2].

Al–Si alloys are highly attractive nonferrous metals in the metallurgy industry due to their advanced performance. These alloys exhibit a high strength-to-density ratio, a high corrosion resistance, good machinability, and a high fluidity and castability. All this makes them highly applicable in the automotive, aerospace, and building industries. Several scientific works have been done on the different effects of the properties of Al–Si alloys [3,4,5,6,7].

Aluminum foam can be produced using different approaches. However, replication methods provide a high repeatability of the produced structure jointly with the ability to control the porosity of the product. For example, Abuserwal et al. reported that effective thermal conductivity decreases with the increase in porosity [8]. Goodall et al. revealed the plasticity size effect. The authors reported that higher strength and rates of hardening are obtained when the structure contains smaller pores [9]. One of the most promising methods of porosity formation is an infiltration process using NaCl as a space holder. This process can be divided into two subprocesses: the first one requires the presintering of the space holder [10], and the second one involves loose bed vacuum infiltration [11]. Using the first approach, a product is totally fit into the presintered template. In this approach, the porosity may be varied by a presintering process that allows a theoretical porosity of 90% to be reached, while a real porosity of up to 75% can be achieved. This technology is relatively costly, and thus, its industrial production is limited. However, the second approach is more economically beneficial and has already been implemented in several industrial companies. A loose bed porosity of 50% can be achieved; however, the application of different compaction methods may lead to a reduction in this value by up to 40% (e.g., a product porosity of 60% can be achieved by application of a monodisperse fraction).

An increase in Al porosity may also be achieved by the application of a space holder mix, made of multisized granules. Kou et al. and Li et al. investigated dual-size and multisize open-cell structures using finite elemental analysis [12,13]. These works focused on the mechanical performance of the fabricated cellular structure and showed a reduction in mechanical properties with an increase in porosity. However, the production of highly porous metallic materials may be beneficial in other functional applications. Thus, their production is applicable in various industries; e.g., porous metals can be used as a heat sink for the cooling of electronics, filtration, and noise reduction, and as aerators, dispersers, and heat exchangers, etc. [14,15,16]. In such applications, an increase in their metallic foam porosity improves their effectiveness. Thus, the focus of the work is the design and fabrication of a highly porous replicated aluminum foam using a double-granular space holder. Here, a double-granular space holder with different granule sizes was used to produce a replicated Al foam. Additionally, a study of the effect of the loose bed parameters on porosity formation was performed, and the granular mix content was evaluated to achieve a maximum compaction.

## 2. Theoretical Principles

The multifraction packing density model was used for the selection of the space holder volume fractions and the ratio of fractions [17,18,19,20,21,22,23]. Packing density models may be divided into geometric, structural elemental scheme, and computer simulation types based on a discrete elemental analysis. Geometric models are devoted to the study of the packing properties of granular materials with space holders of various granular ranges and consider compaction, wall (porosity decreases near the wall), and loosening effects (coarse granules separation with the introduction of fine granules). Modeling of the porosity process allows for the prediction of the porosity of the structure; however, this is a complicated approach that requires special software and time for the simulation.

The determination of the optimal ratios of the volumes and granular mixes has been done using the geometric model of De Larrard [23], which considers all the above-mentioned factors. The basic equation of the model for the packing of the double-granular mixes is:

(1) $$\frac{\frac{X_{1}}{\beta_{1}}}{\frac{1}{\alpha }-\frac{1}{\gamma_{1}}}+\frac{\frac{X_{2}}{\beta_{2}}}{\frac{1}{\alpha }-\frac{1}{\gamma_{2}}}=K$$

where $X_{i}$ is the volume of the *i-th* monodisperse fraction in the mix (the same density of both granules leads to equality in mass fraction), α is the actual density of the double packing, $\beta_{i}$ is the virtual packing density of the *i-th* monodisperse fracture in the mix, $\gamma_{i}$ is the virtual packing density of the *i-th* monodisperse fracture in the mix when *i* is the dominant fracture, K is the compaction factor, which depends on the packing process, and *i* = 1, 2 are different granules fractures with the granular diameters $d_{1}\;$ > $d_{2}$.

The virtual packing density is a key component of the model; this is the maximum packing density with the ideal granular occupancy. For example, for the packing of the monodisperse soil, the virtual packing factor is equal to 0.74, whereas the actual packing factor varies from 0.60 to 0.64, depending on the compaction method.

The value of $\beta_{i}$ is evaluated by using the actual packing density of the monodisperse fracture:

(2) $$\beta_{i}=\alpha_{i}\left(1+\frac{1}{K}\right)$$

where the compaction index K is dependent on the packing process in accordance with the values in Table 1.

The value of $\gamma_{i}$ is evaluated using the following equations:

(3) $$\gamma_{1}=\frac{\beta_{1}}{1-\left(1-a_{12}\frac{\beta_{1}}{\beta_{2}}\right)X_{2}}$$

(4) $$\gamma_{2}=\frac{\beta_{2}}{1-\left(1-b_{21}\beta_{2}\left(1-\frac{1}{\beta_{1}}\right)\right)X_{1}}$$

where $a_{12}$ and $b_{21}$ are empirical values that refer to the loosening and wall effects, respectively:

(5) $$a_{12}=\sqrt{1-{\left(1-\frac{d_{2}}{d_{1}}\right)}^{1.02}}$$

(6) $$b_{21}=1-{\left(1-\frac{d_{2}}{d_{1}}\right)}^{1.5}$$

**Figure 1:**
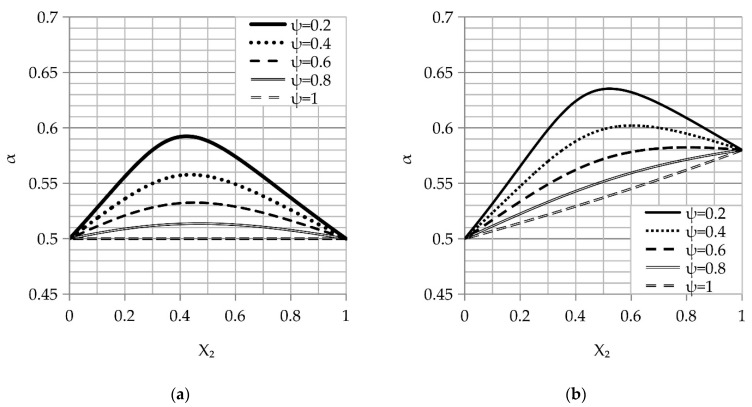
Plots of the actual density of the double fracture packing (α) as a function of the second fracture (X_2_) content in the granular mix (ψ =$\;d_{2}/d_{1})$ at the same packing process (pouring compaction index (K = 4.1) for: (**a**) the same packing density of the monodisperse granules and (**b**) the different packing density of the monodisperse granules.

The main aim of this work is to determine the granular fraction ratios, their size in both fractions, and the maximum compaction necessary to achieve the highest packing density. The De Larrard model has been applied to analyze the variation in the actual density of the packing as a function of the second fraction content X_2_ in the granular mix (Figure 1).

The analysis of the curves shown in Figure 1 revealed that the maximum packing density increases with the increase in the granular fraction ratio. As shown in Figure 1a, the maximum is reached when X_2_ is in the range of 0.4–0.5 for the same packing density ($\alpha_{2}=\alpha_{1}\;\left(\beta_{2}=\beta_{1}\right)$) and the maximum increases slightly with higher values for the different packing densities ($\alpha_{2}>\alpha_{1}\;(\beta_{2}>\beta_{1}))$, as addressed in Figure 1b.

**Figure 2:**
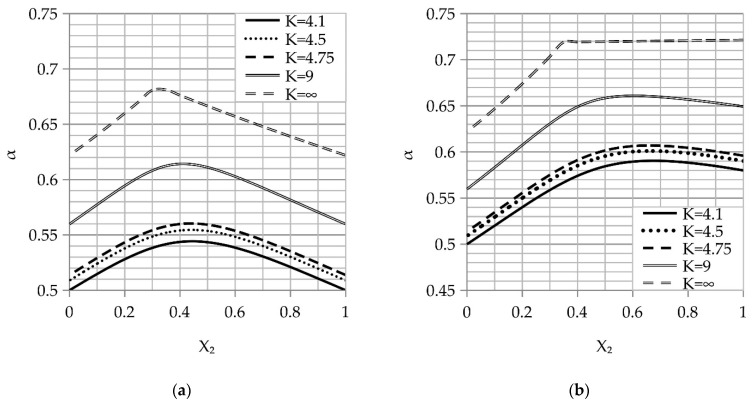
The plots of the actual density of the double fracture packing (α) as a function of the second fraction content (X_2_) in different packing processes (K) at the same granular mix (ψ = 0.5) for: (**a**) the same packing density of the monodisperse fractures and (**b**) the different packing density of the monodisperse fractures.

Figure 2 shows the effect of the compaction index K on the actual density of the packing as a function of the second fraction content X_2_ in the granular mix at a constant granule fracture (ψ = 0.5).

Figure 2a reveals that the maximum packing density is reached when the X_2_ values are in the range of 0.4–0.5 for the same packing density. However, evaluation of Figure 2b reveals that the maximum packing density may be achieved when the X_2_ values are greater than 0.5 for the different packing densities of the monodisperse granules.

The aim of the present work is the design and production of a replicated aluminum foam with enhanced porosity. First, for the space holder at room temperature, the De Larrard model was verified. Then, different methods comparing the effects of the space holder compaction on the porosity formation were investigated.

## 3. Materials and Methods

The highly porous replicated aluminum foam was synthesized over three stages. At the first stage, the actual porosity of the monodispersed space holder was determined. At the second stage, the double fraction loosening was investigated. At the final stage, porous Al foam samples were produced and investigated. In the work, compaction methods applicable for a hot space holder were investigated.

Stage 1.

**Figure 3:**
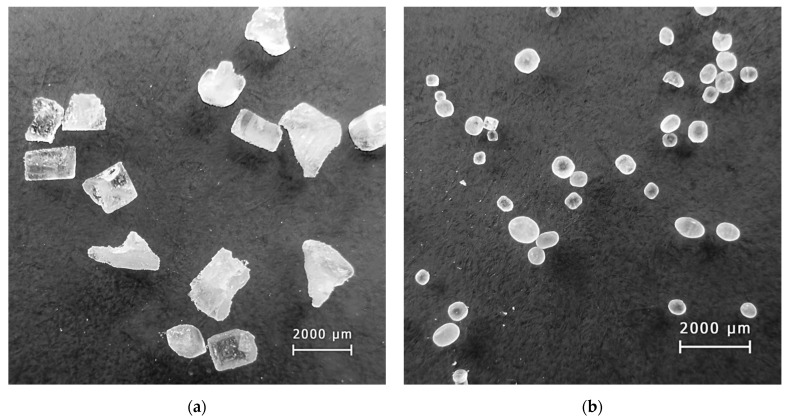
A space holder powder of sodium chloride used in the work: (**a**) irregular shaped and (**b**) spherical shaped.

Sodium chloride (NaCl) granular mixes with different shapes were used as a space holder. Spherical granules (Mozyrsalt, Mozyr, Belarus) are illustrated in Figure 3a and irregular granules (Iletsksol, Sol-Iletsk, Russia) are illustrated in Figure 3b. The sieving of the space holder was done using the sieve shaker LPzE-2e (MULTISERW-Morek, Brzeźnica, Poland). The fraction nomination used in the work is shown in Table 2.

The determination of the actual packing density for different packing processes was evaluated using measurements of the mass with a laboratory mass balance CE-6202C (SARTOGOSM, Saint Petersburg, Russia). The measurements were made using the following equation:

(7) $$\alpha_{i}=\frac{m}{\rho V}$$

where m is the mass of the measured substance, V is the volume of the measured substance, and ρ=2163 kg/m^3^ is the density of the NaCl space holder [24].

The actual packing density was assessed using the following compaction methods: pouring without compaction, manual ramming followed by ten impacts, and vibration for 10 s. The average values of five measurements are presented in the results.

Stage 2.

An additional fraction of the space holder was added and manually mixed into a portion of the main fracture and calculated for the total mass of the mix of 400 g. The additional space holder fractions were 0.2, 0.3, 0.4, 0.5, 0.6, 0.7, and 0.8. The packing density was measured as described in stage 1.

Stage 3.

Porous Al foam samples were obtained using vacuum casting as described in [25]. AlSi7 alloy (LLC Uraltsvetlit, Kamensk-Uralsky, Russia) at 710 °C was poured into a specially designed cylindrical metal cast with a diameter of 52 mm and a height of 170 mm. The cast was preheated at 350 °C and filled with a granular mix. The mix was preheated at 630 °C followed by compaction with ten impacts and manual ramming. Four samples were poured in each experiment. The additional space holder fractions were 0.2, 0.3, 0.4, 0.5, 0.6, 0.7, and 0.8.

Samples for macrostructural examination were cut from the obtained casts with a diameter of 45 mm and a height of 10 mm. The space holder was dissolved in water and cut for the macrostructural evaluation, which was carried out using a GX51 (Olympus, Tokyo, Japan).

## 4. Results and Discussion

**Figure 4:**
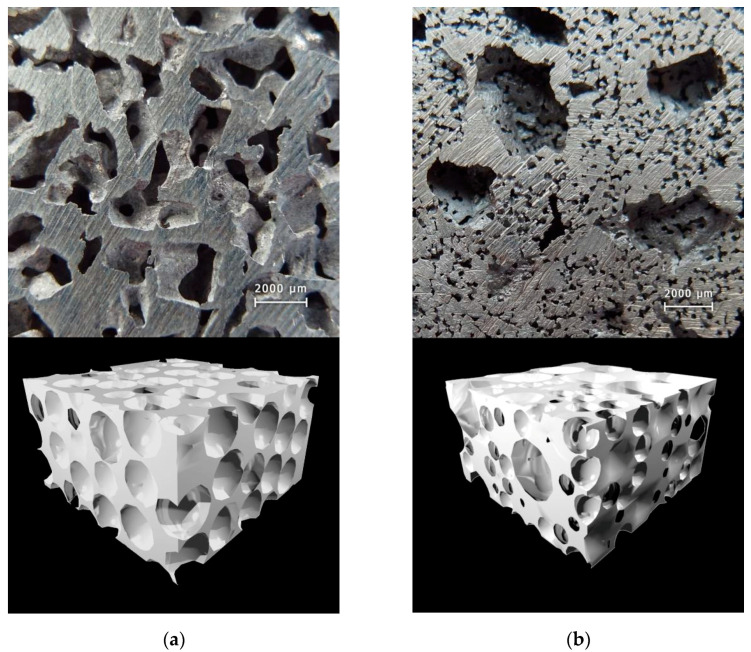
The macrostructure and corresponding 3D model of Al replicated foam produced using: (**a**) a monodispersed space holder and (**b**) a double-granular space holder.

The macrostructure of the replicated Al foams produced using monodispersed and double-granular space holders and corresponding 3D models are shown in Figure 4a,b, respectively.

The measured values of the actual and virtual packing densities of the monodispersed granules are shown in Table 3.

The results revealed from Table 3 point to the high degree of dependence of the packing density on the shape of the granules. Spherical granules exhibit roughly 15% higher values for actual and virtual packing. This phenomenon may be attributed to the shape of the granules, as spherical granules exhibit roundness and a smooth surface thereby producing denser packing [26].

**Figure 5:**
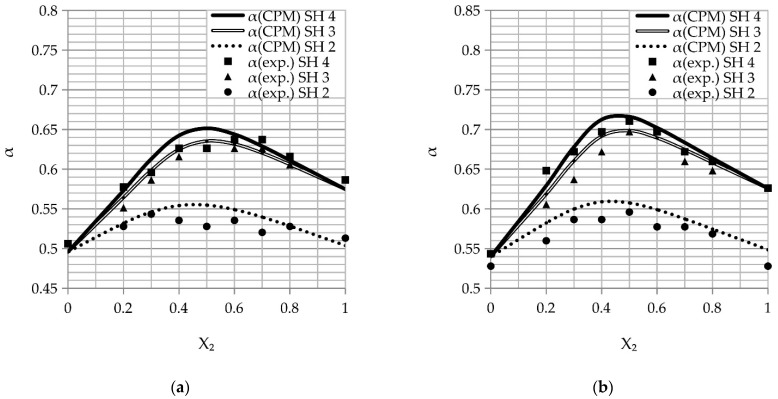
The plots of an experiment calculated by the De Larrard model showing the actual packing density of the double fracture (α) as a function of the second fracture (X_2_) content in different packing processes: (**a**) pouring and (**b**) 10 impacts with a rod. Exp—experimental values, CPM—compaction packing model (De Larrard model).

The experimental values for the double fracture loosening are shown in Figure 5. These curves also present values for the double fracture loosening calculated from the De Larrard model. Here, values of the compaction index were corrected due to differences in the compaction of the actual mixes compared to the theoretical. Thus, when any compaction method is applied, granules with sharp edges move and partially destroy other granules. Moreover, the compaction values listed in Table 1 were determined using mixes related to concrete, which are much harder in comparison with the sodium chloride mixes used in this work. Therefore, the compaction indexes K were increased; the following values were corrected and implemented in calculations: 3.7 for the pouring (Figure 5a), and 6 for 10 impacts with a rod (Figure 5b).

An examination of the curves in Figure 5 showed that the most effective compaction method is vibration. However, this method is mostly applied in industrial applications for large-scale products. Thus, the most effective method to produce small-size products is impact with a rod.

**Figure 6:**
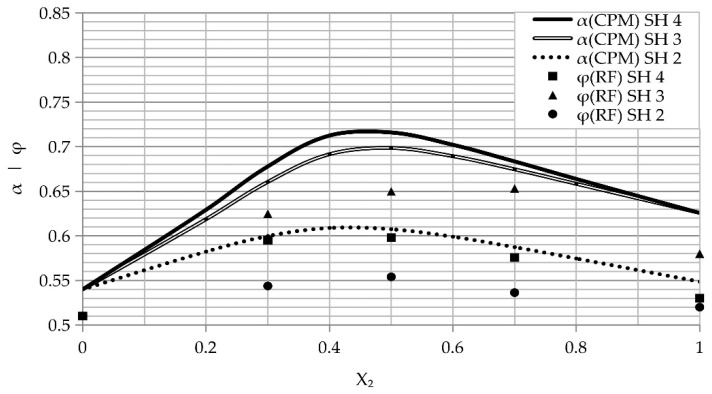
The curves of the porosity (ϕ) and an actual packing density of the double fracture packing (α) as a function of the second fracture (X_2_) content for different sizes of granules are shown. RF—replicated foam, CPM—compaction packing model (De Larrard model).

The obtained Al foam porosity is shown in Figure 6. This plot also contains the actual density of the NaCl space holder at room temperature, in accordance with the De Larrard model. The presented curves address the different sizes of the granules.

An evaluation of the curves in Figure 6 revealed that the aluminum foam’s porosity does not reach the values of the actual packing density without presintering. This behavior points to a low efficiency of the space holder compaction and may be attributed to the diffusion processes of the space holder during the presintering treatment [10]. It is most dominant in a finer fracture of the space holder, resulting in a lower gap between the mix granules. An evaluation of the curves revealed that the application of a double-granular space holder led to an increase in the porosity of the Al replicated foam. A maximum porosity of 65% was obtained via the compaction method using 10 impacts with a mix of space holder SH 1 (irregular granules) and 50–70 wt. % SH 3 (spherical granules). It is logical to assume that the application of finer granules to the mix should increase the porosity of the product. However, as determined in the curves of Figure 6, this behavior was not achieved.

**Figure 7:**
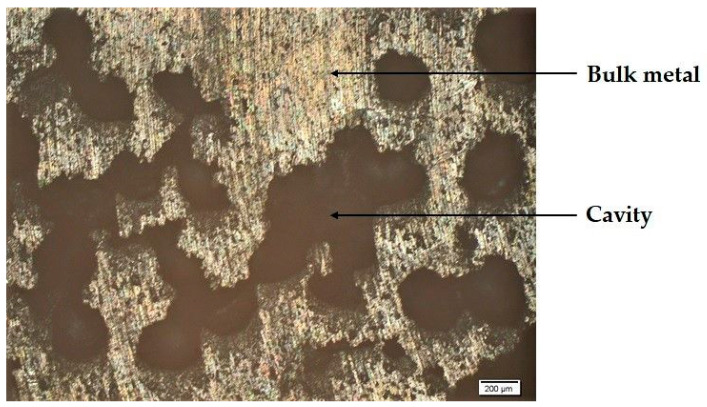
The macrostructure of a highly porous replicated Al foam obtained using a double-granular space holder. The space holder is a mix of SH 1 coarse, irregular granules and SH 4 fine, spherical granules.

To understand the reason for such behavior, the replicated Al foam macrostructure obtained in the compaction method using 10 impacts on the mix of SH 1 coarse granules and fine SH 4 granules was investigated as shown in Figure 7.

An evaluation of the macrostructure in Figure 7 revealed the presence of large cavities formed as the result of fine granules sintering into the mix. These cavities were partially filled by bulk metal, contributing to the formation of a less porous product. As a result of the fine granules sintering, their fracture into the mix reduced the fine granules to a level where they have no effect on the porosity. Therefore, it may be concluded that the application of very fine granules is disadvantageous for porous replicated Al foam formation.

The pore size distribution of the replicated Al foam obtained by the loose bed using a monodispersed fracture of the space holder may be determined by the bottleneck between its granules [22]. However, the estimation of the pore size distribution using a double-granular space holder is a more complicated task, which should be solved separately. It should consider minimum pore size by bottlenecks between two small granules, one large and one small granule, and two large granules. Concomitantly, maximum pore size may be estimated by the dominant and additional fractures of the space holder.

## 5. Conclusions

In this work, a highly porous replicated Al foam using a double-granular space holder was designed based on the De Larrard model of multifunctional packing density. The model was corrected and the optimal parameters to obtain the maximum porosity were evaluated. The calculations showed an expected behavior whereby spherical granules exhibited 15% higher packing values compared to irregular-shaped granules. This phenomenon was attributed to the granules’ roundness and smooth surface. The model of multifunctional packing density was also used to estimate compaction values for the replicated Al foam fabrication. Then, several mixes that contained coarse, irregular sodium chloride space holders with different fractures of fine space holders were produced. The results pointed to the highest porosity of 65% achieved with the mix of coarse, irregular granules of 2500–4000 µm and fine, spherical granules of 400–630 µm. The use of finer granules as space holders was found to be disadvantageous. Their application led to cavity formations followed by their refilling with Al bulk. These cavities were formed as the result of the dissolution of several sintered, fine NaCl granules that formed one coarse granule.

## Figures and Tables

**Table 1:** Values of compaction index K assigned to different packing processes [23].

Packing Process	Compaction Index (*K*)
Pouring	4.1
Impacts with a rod	4.5
Vibration	4.75
Vibration + compression (10 kPA)	9

**Table 2:** Values of compaction index (K) addressed to different packing processes. SH—space holder.

Granular Fracture	Granular Size (µm)	Granular Shape
SH 1	2500–4000	irregular
SH 2	1000–1600	irregular
SH 3	400–600	spherical
SH 4	200–400	spherical

**Table 3:** Values of actual and virtual packing densities for different monodispersed granules.

Granular Fracture	Actual Packing Density (α)	Virtual Packing Density (β)
SH 1	0.506 ± 0.005	0.629
SH 2	0.513 ± 0.006	0.638
SH 3	0.587 ± 0.005	0.730
SH 4	0.587 ± 0.008	0.730

## Data Availability

The data presented in this study are available on request from the corresponding author.
